# The “springform” technique in cranioplasty: custom made 3D-printed templates for intraoperative modelling of polymethylmethacrylate cranial implants

**DOI:** 10.1007/s00701-021-05077-7

**Published:** 2021-12-06

**Authors:** Johannes P. Pöppe, Mathias Spendel, Christoph Schwartz, Peter A. Winkler, Jörn Wittig

**Affiliations:** 1 Department of Neurosurgery, University Hospital Salzburg, Paracelsus Medical University, Ignaz-Harrer-Str. 79, 5020 Salzburg, Austria; 2 Department of Oral and Maxillofacial Surgery, University Hospital Salzburg, Paracelsus Medical University, Müllner Hauptstraße 48, 5020 Salzburg, Austria

**Keywords:** 3D printing, Cranioplasty, Patient-specific implants, Stereolithography, Sterilisable templates, Springform technique

## Abstract

**Background:**

Manual moulding of cranioplasty implants after craniectomy is feasible, but does not always yield satisfying cosmetic results. In contrast, 3D printing can provide precise templates for intraoperative moulding of polymethylmethacrylate (PMMA) implants in cranioplasty. Here, we present a novel and easily implementable 3D printing workflow to produce patient-specific, sterilisable templates for PMMA implant moulding in cranioplastic neurosurgery.

**Methods:**

3D printable templates of patients with large skull defects before and after craniectomy were designed virtually from cranial CT scans. Both templates — a mould to reconstruct the outer skull shape and a ring representing the craniectomy defect margins — were printed on a desktop 3D printer with biocompatible photopolymer resins and sterilised after curing. Implant moulding and implantation were then performed intraoperatively using the templates. Clinical and radiological data were retrospectively analysed.

**Results:**

Sixteen PMMA implants were performed on 14 consecutive patients within a time span of 10 months. The median defect size was 83.4 cm^2^ (range 57.8–120.1 cm^2^). Median age was 51 (range 21–80) years, and median operating time was 82.5 (range 52–152) min. No intraoperative complications occurred; PMMA moulding was uneventful and all implants fitted well into craniectomy defects. Excellent skull reconstruction could be confirmed in all postoperative computed tomography (CT) scans. In three (21.4%) patients with distinct risk factors for postoperative haematoma, revision surgery for epidural haematoma had to be performed. No surgery-related mortality or new and permanent neurologic deficits were recorded.

**Conclusion:**

Our novel 3D printing-aided moulding workflow for elective cranioplasty with patient-specific PMMA implants proved to be an easily implementable alternative to solely manual implant moulding. The “springform” principle, focusing on reconstruction of the precraniectomy skull shape and perfect closure of the craniectomy defect, was feasible and showed excellent cosmetic results. The proposed method combines the precision and cosmetic advantages of computer-aided design (CAD) implants with the cost-effectiveness of manually moulded PMMA implants.

**Supplementary Information:**

The online version contains supplementary material available at 10.1007/s00701-021-05077-7.

## Introduction

Cranioplasty in patients with large skull defects, e.g. after decompressive hemicraniectomy, is regularly performed for mechanical, cosmetic and physiological reasons [7, 17, 29]. Main physiological reasons are to restore the intracranial pressure equilibrium and thus improve cerebral blood flow, cerebral perfusion and probably improve neurological outcome [7, 17, 29]. A great variety of implants have been established, primarily cryopreserved autologous bone grafts, computer-aided design (CAD) implants and intraoperative modelling of formable thermoplastic polymers like polymethylmethacrylate (PMMA) [9]. However, an international standard for which material should be preferably used has not yet been established [4].

All materials for cranioplasty have certain advantages and disadvantages: CAD implants are expensive and are time consuming to produce because of industrial manufacturing; Autologous implants tend to have higher reoperation rates mainly due to bone resorption, whereas infection rates are lower compared to alloplastic grafts [11, 19, 30, 31]; intraoperative manual modelling of PMMA implants is — in our experience — inferior to autologous implants and CAD implants from a cosmetic standpoint. Advantageous aspects of PMMA are that it is widely used and thoroughly tested for cranial reconstruction surgery [4, 9], can be easily formed as a dough and cures within several minutes through a polymerisation process.

Furthermore, at our institution, intraoperative microbiological testing during craniectomy revealed significant contamination primarily with *Propionibacterium acnes* and *Staphylococcus epidermidis*; thus, we had to discard almost 50% of all explanted autologous bone grafts due to national government regulations from 2018 on.

In recent years, 3D printing has become a commonly used tool in neurosurgery [21, 27], with a number of practical applications such as anatomic models [28], surgical education and operation planning [15, 16, 18]. Several ideas have been tested using 3D-printed moulds for intraoperative or preoperative formation of custom implants for cranioplasty [1–3, 5, 10, 13, 14, 23, 25, 26], even for posterior fossa reconstructions [20]. Two main strategies have been applied: first, to 3D print a virtually designed implant and, using this as a template, to produce sterilisable silicon moulds from which the PMMA implant can be formed intraoperatively [2, 5, 23]. Similar approaches have also been described with the original bone flap as a template for moulding [12]. Second, to 3D print one or two moulds, which represent the inner and/or outer curvature of the planned implant. In a model with one mould [25], PMMA is manually adapted to the template, whereas in models with two moulds, the PMMA is pressed between the two moulds to form the implant [1, 3, 10, 13, 14, 26].

Based on these existing concepts, we invented a “springform-like” moulding technique, to simplify and improve the planning and moulding process. Unlike in other procedures, no additional material is required, such as sterile plastic bags to cover unsterile moulds [1] or silicon moulds that are produced from a 3D-printed implant [2, 5, 23]. This reduces the expense of implant production and rules out additional sources of error.

Our approach concentrates on precise reconstruction of *outer* cranial shape and optimised closure of the bone defect whilst modelling the *inner* curvature by hand. The inner surface seems to be physiologically — and of course cosmetically — less critical, for which thin titanium mesh implants with comparable complication rates to other allografts are a comprehensible proof [22]. This way, instead of a second mould, as described elsewhere [1, 3, 10, 13, 26], only a template ring is required, saving printing time and printing material. Furthermore, the individual thickening of the implant can be adapted to residual brain swelling or a thick layer of epidural scar tissue, which can be left in place avoiding incidental dural tears. Instead of applying mirroring of the contralateral skull shape or digital modelling to design implant shape — which has been described in several publications [1, 2, 5, 10, 14, 20, 23, 26] — we use pre craniectomy CT scans for skull reconstruction whenever possible. This not only supports optimal reshaping of the patient’s skull, but also saves time and simplifies the planning process.

We aimed to develop and clinically implement a 3D printing workflow to produce sterilisable, patient specific, 3D-printed templates for intraoperative manufacturing of cranial PMMA implants within our institution. Here, we present our clinical and radiological results.

## Methods

### Template design

**Fig. 1:**
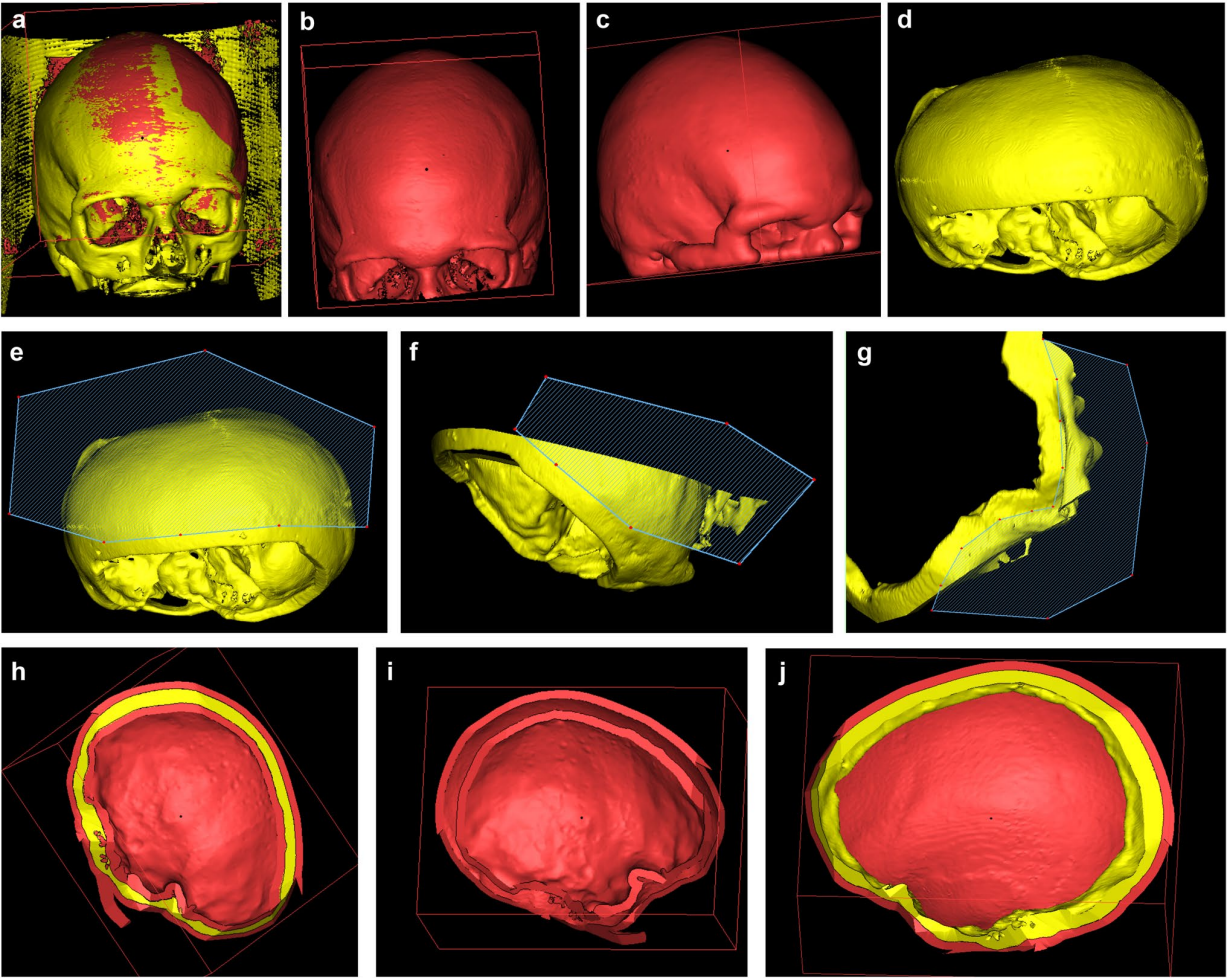
Template design in Materialise Mimics InPrint software in chronological sequence: Import of fused precraniectomy (red) and postcraniectomy (yellow) (PreCE/PosCE) reconstructions of the skull (**a**), hollowing of PreCE model (**b** **c**) and cutting of both, PreCE and PosCE model leaving a ring of 10–15 mm around the defect (**d**–**g**). This results in a two-layered hollow of PreCE (**h**, **i**). The inner hollow is deleted, resulting in final templates — a ring of PosCE and a cover of PreCE (**j**)

**Fig. 2:**
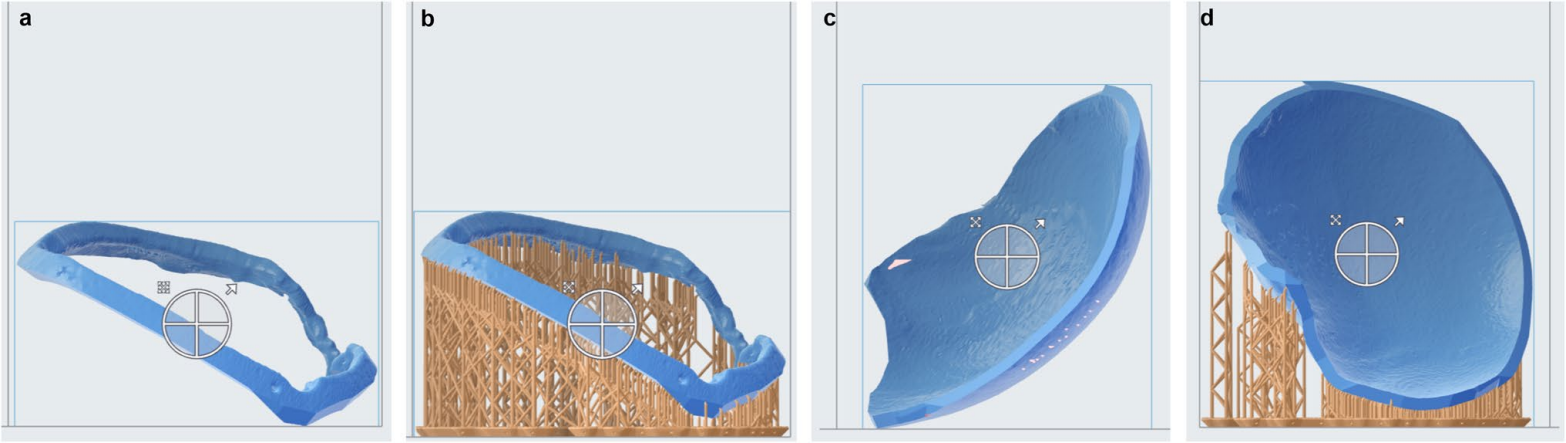
Ring (**a**, **b**) and cover (**c**, **d**) template are loaded into PreForm 3.12.2 software and support structures for printing are automatically added (**b**, **d**). No support structures are added at the inner side of the cover and the outer side of the ring template, thus ensuring an even surface for implant moulding

Computed tomography (CT) images with a slice thickness of 1 mm from patients prior to, and after craniectomies, were fused using Brainlab iPlan 3.0 (Brainlab AG, Munich, Germany) software. Semiautomatic threshold segmentation excluding soft tissue was performed. This way, 3D objects of the bony skull before and after craniectomy were created and loaded as stereolithography (STL) files into the Materialise Mimics inPrint 3.0 software (Materialise Inc., Leuven, Belgium) (Fig. 1a). Both the Brainlab iPlan and Materialise Mimics software are registered as CE-certified class I medical devices in cranio-facial surgery. Extracranial structures with high density (such as wound staples) that were part of the 3D model could be eliminated using the isolation function of the software. The precraniectomy model (PreCE) was then hollowed with a layer thickness of 5 mm (Fig. 1b–c). The resulting hollow (Fig. 1c) and the postcraniectomy model (PosCE, Fig. 1d) were cut along the craniectomy defect, leaving a rim of approximately 10 mm around the defect (Fig. 1e–g). Afterwards, the inner hollow was deleted leaving the outer layer as a mould of the PreCE skull and the rim as a representation of the PosCE which fits like a ring precisely into the outer hollow (Fig. 1h–j). Both templates were imported as STL files into the printer software PreForm 3.12.2 (Formlabs Inc., Somerville, MA, USA) and support structures for printing were added (Fig. 2).

### 3D printing

Both templates (mould and ring) were printed separately on a Formlabs Form 3B or Form 2 stereolithography printer using biocompatible photopolymer resins (Formlabs Dental SG Resin, Formlabs Surgical Guide Resin). A layer resolution of 0.1 mm was applied. Both resins are CE certified as medical devices class I for printing of surgical guides in dentistry and can be sterilised. Afterwards, printed support structures were removed. Wash and cure requirements were completed, followed by sterilisation with chemical disinfection (70% isopropyl alcohol for 5 min) and steam sterilisation (autoclave at 134 °C for at least 20 min) in our in-hospital sterilisation unit, as prescribed by the company (Formlabs Material Data Sheet).

### Virtual surgery simulation

Prior to the first surgical application of the method, a trial implant was produced in the described manner. The heating of PMMA during polymerisation process did not affect the shape or structure of the cured resin templates. A CT scan of the PMMA implant was performed. Virtual implantation, via fusion with the postcraniectomy CT of the same patient in Brainlab iPlan 3.0 software, confirmed excellent fit and shape. Thus, major deviations in size and shaping of the implant throughout the design and printing process could be ruled out.

### Implant creation

**Fig. 3:**
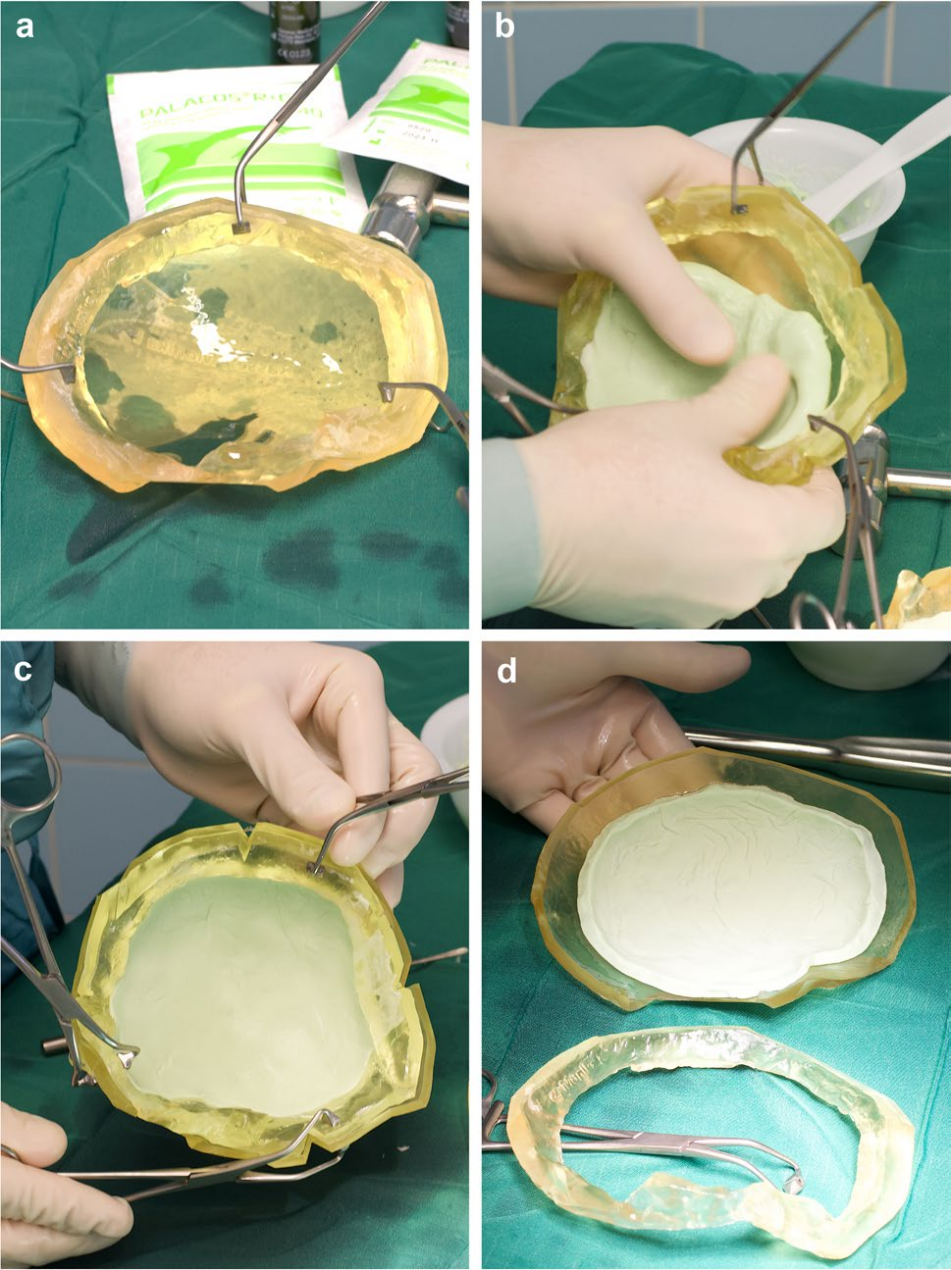
Preoperative implant production: ring and cover template are clamped firmly together, a thin layer of neutral oil is applied (**a**). PMMA dough is modelled into the templates until heating occurs (**b**). After largely completed polymerisation, the ring is removed (**c**, **d**) and the implant can be taken from the cover

**Fig. 4:**
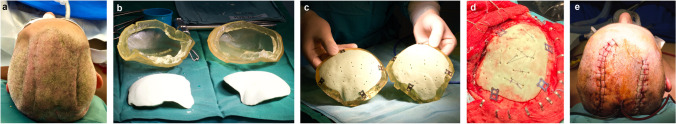
Illustrative case in chronological order: preoperative skin after bilateral decompressive CE (**a**), production of both implants preoperatively (**b**), placement of fixation plates and mini burr holes for retention sutures (**c**), implant fixation into the defect (**d**), postoperative skin curvature (**e**)

Intraoperative production of the cranial implant was performed in most cases directly before skin incision under sterile circumstances in the operating room. This is demonstrated in Fig. 3 and Video 1:

1. The templates were attached with surgical cover clamps and covered with a thin layer of neutral oil (Fig. 3a).
2. Gentamicin containing PMMA dough (Palacos R + G, Heraeus Medical, Wehrheim, Germany) was evenly moulded into the templates with consideration of anatomical conditions such as the thinner wall of the temporal bone. The amount of PMMA was chosen by the operating surgeon: approximately 60 ml PMMA was used for large decompressive craniectomy defects (Fig. 3b).
3. Following the heating of the polymerisation process, the template ring could be removed and overlapping PMMA material was cut away (Fig. 3c–d).
4. After complete polymerisation, the implant was withdrawn from the outer mould and checked for sufficient fit. If necessary, the edges were adjusted with a high-speed drill. Perforating drill holes were generously placed and four four-hole plates already affixed using the 3D-printed ring as a template (Fig. 4c).

### Cranioplasty surgery

The implantation surgery was performed in accordance with institutional standards. Skin opening was performed along the previous incision, followed by preparation of bony margins, (neo-)dura and a temporalis muscle flap. Implants were fixed with four four-hole plates and self-tapping titanium screws (Fig. 4c–d). The dura was attached to the implant via multiple resorbable retention sutures to prevent epidural fluid collection. One or two subgaleal drains with suction were inserted, followed by wound closure. A cranial CT scan was regularly performed the following day. A postoperative follow-up after discharge was routinely conducted after 2 to 4 weeks at our outpatient clinic.

### Patient selection and population

In this case series, fourteen consecutive patients (9 males, 5 females; median age 51 years, range 21–80 years) with large craniectomy defects for whom no autologous implant was available were treated from June 2020 to April 2021 at our institution with the above described cranioplasty technique. All analysed preoperative and postoperative clinical data were acquired retrospectively from electronic patient records.

## Results

Clinical characteristics of the cohort are shown in Table 1. A total of 16 cranial implants were performed: five left and six right sided fronto-temporo-parietal (FTP) defects, one left sided fronto-temporal defect and two bilateral FTP defects. The median size of the defects was 83.4 (range 57.8–120.1) cm^2^. The indications for craniectomy were traumatic brain injury with subdural haematoma (*n* = 4), ischemic stroke (*n* = 4), intracerebral haemorrhage (*n* = 3), delayed cerebral ischemia in subarachnoid haemorrhage (*n* = 1), drug-induced generalised brain edema (*n* = 1) and postoperative empyema after meningioma resection (*n* = 1). The median time to cranioplasty after initial bone flap removal was 80 (range 10–192) days.

**Table 1:** Patients who underwent cranioplasty with “springform” technique 3D-printed templates for moulding of PMMA implants at Christian-Doppler-Hospital Salzburg between June 2020 and April 2021 (patients with bilateral cranioplasties were divided into cases a and b respectively)

Patient ID	Sex	OR time (min)	Time to CP (days)	Age at CP	Indication for craniectomy	Location	Max. diameter ap (mm)	Max. diameter cc (mm)	Defect dimension cm^2^	Complications
1	m	152	58	21	Right temp. ICH, dAVF	R FT	127	91	57.8	None
2	f	105	114	36	Malignant ACM stroke	R FTP	133	128	85.1	None
3	f	81	78	53	Epidural Empyema after meningeoma resection	L FT	120	101	60.6	None
4a	m	105	112	21	Generalised brain edema, multiple ischemic lesions (drug induced)	L FTP	136	102	69.4	None
4b	m	52	112	21	„	R FTP	139	126	87.6	None
5	m	136	82	68	TBI, aSDH	L FTP	120	120	72	None
6a	f	84	149	43	Left post. ICH & AVM surgery	L FTP	134	122	81.7	EDH
6b	f	60	149	43	„	R FTP	98	123	60.3	None
7	m	80	53	52	Malignant ACM stroke	L FTP	138	124	85.6	EDH
8	m	75	10	80	TBI, aSDH	R, FTP	117	115	67.3	None
9	m	76	18	56	TBI, aSDH, frontal skull fracture	R FTP	163	130	105.95	None
10	m	112	192	65	ICH	R FTP	133	96	63.8	None
11	m	75	166	50	Malignant ACM stroke	R, FTP	150	127	95.25	EDH
12	f	65	18	52	TBI, aSDH	L FTP	165	139	114.7	None
13	m	90	112	43	Malignant ACM stroke	L FTP	152	147	111.7	Subgaleal fluid collection
14	f	137	70	38	Malignant ACM stroke	L FTP	155	155	120.1	None

Abbreviations:* ID* identity, *m* male, *f* female, *OR* operating room, *CP* cranioplasty, *ap* anterio-posterior, *cc* cranio-caudal, *ICH* intracerebral haemorrhage, *dAVF* dural arterio-venous-fistula, *ACM* arteria cerebri media, *TBI* traumatic brain injury, *aSDH* acute subdural hematoma, *FT* fronto-temporal, *FTP* fronto-temporo-parietal, *L* left, *R* right

**Fig. 5:**
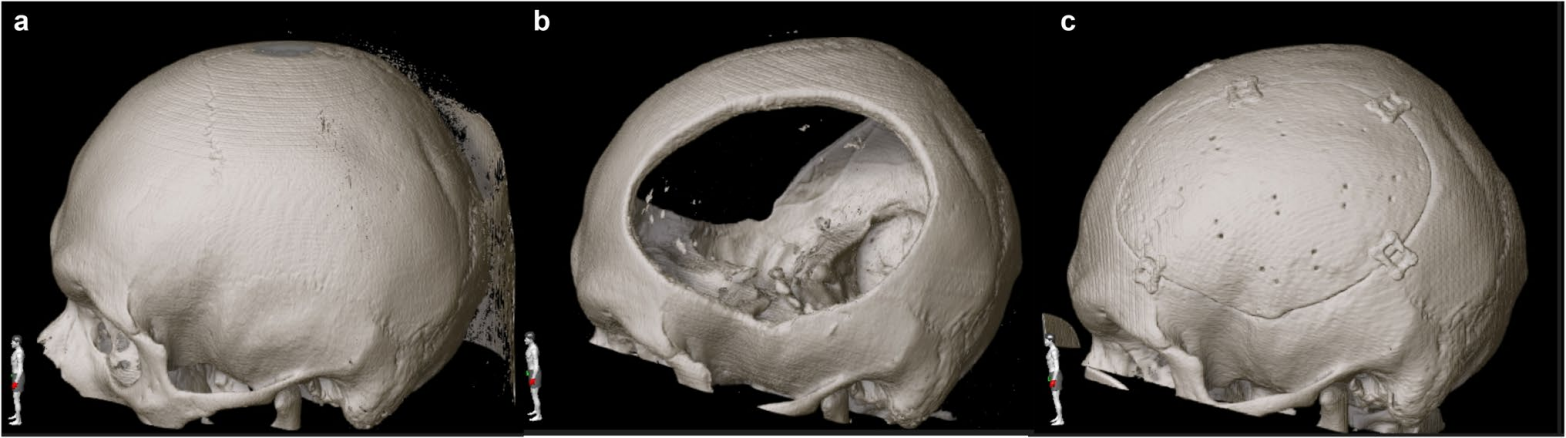
3D reconstruction of cranial CT scans before craniectomy (**a**), after craniectomy (**b**) and after cranioplasty (**c**) in a patient with bilateral decompressive craniectomy

**Fig. 6:**
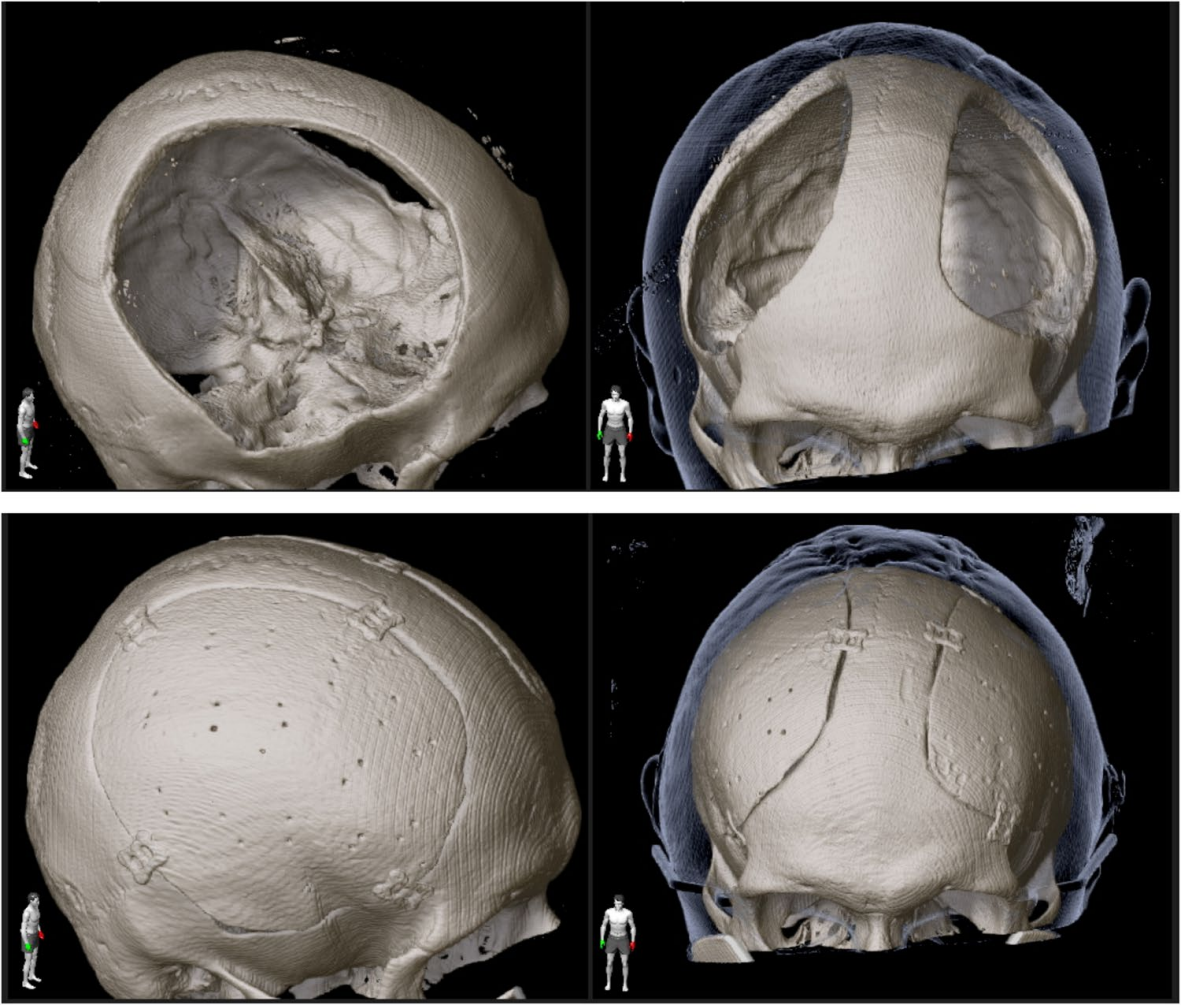
Comparison of prebilateral cranioplasty (upper row) and postbilateral cranioplasty (lower row) shows very good fit of the implants and symmetrical reshaping of the skull

Intraoperatively, all implants fitted well into the craniectomy defects, and only minor alterations, made with a high-speed drill, were deemed necessary. No manual modelling had to be applied as an alternative and no intraoperative complications occurred. Postoperative CT scans revealed excellent reconstruction of the cranial shape compared to precraniectomy scans in all patients (Figs. 5 and 6). The median skin-to-skin operating time per implant was 82.5 (range 52–152) min.

Average costs on the basis of an estimated number of 30 implants per year in our institution were calculated to be about 300€ (= 360 US $) per implant. This includes costs for the 3D printer over an amortisation period of 5 years, additional software licenses and expendable material such as resins and printer parts — but not staff working hours.

### Complications and clinical outcome

In three (21.4%) patients, a postoperative intracranial haemorrhage, requiring revision surgery, occurred. In two of those patients, an epidural haematoma was revealed on routine CT scans at first postoperative day (IDs 7 and 11). Both patients had significant clinical risk factors for the development of intracranial bleeding. Patient 11 had a strict indication for anticoagulation because of a mechanical mitral valve implant. Preoperative bridging therapy was performed with low molecular weight heparin, which was paused on the day of the surgery. The patient received continuous intravenous heparin infusion therapy 4 h postoperatively, only after a CT scan had ruled out intracranial bleeding. Patient 7 received a ventriculo-peritoneal shunt because of posthaemorrhagic hydrocephalus in the same anaesthesia directly prior to cranioplasty. Even though both patients showed no specific clinical symptoms, revision surgery was performed due to the space-occupying effect (> 10 mm) of the haematoma. The implants were not exchanged. In follow-up CT scans no relevant epidural or subdural bleedings were observed. In another patient (ID 6) with a bilateral cranioplasty, a symptomatic epidural haematoma occurred on the left side on the second postoperative day, after a routine CT scan on the first day had shown no signs of intracranial bleeding. Further clinical workup revealed reduced Factor XIII (fibrin stabilizing factor) levels of only 55%, which provide a clinical explanation for the delayed postoperative bleeding. Revision surgery was performed and Factor XIII substituted. No further complications occurred in clinical follow-up. In one patient (ID 13), a subcutaneous fluid collection occurred, most probably because of a cerebrospinal fluid leakage. The fluid had to be drained with transcutaneous puncture, but no signs of infection were revealed in laboratory workup and no revision surgery was needed. No surgery-related mortality or new and permanent neurologic deficits were recorded. No postoperative infections or wound healing deficits were recorded.

In postoperative clinical follow-up, all patients/legal representatives were satisfied with skull shape and cosmetic appearance.

## Discussion

The novel “springform” technique for cranioplasty is feasible and combines the advantages of in-house production with the quality of CAD implants at much lower costs. All implants showed excellent fit and appealing cosmetic results. Operation time was short and intraoperative handling of the moulding process straightforward. Thus, we were able to implement the described workflow in our institution. In our series, excellent cosmetic results could be achieved which are in line with other 3D printing studies for implant moulding in cranioplasty [6, 13].

The proposed design technique can be performed by surgeons or trained medical personnel. To perform all steps of implant creation in one institution — from image fusion and segmentation to printing and sterilisation — gives the surgeon the possibility to guarantee quality throughout the whole process and apply changes in design as needed. Growing experience with the technique directly leads to improvement of the design process in the very sense of translational medicine. This principle seems to us, especially from a scientific view point, an improvement compared to industrial CAD implants or 3D-printed moulds that are manufactured externally [3, 13].

### Operating time

A reasonable aim of improving cranioplasty surgery is to reduce the net operating time and thus, time under anaesthesia and wound exposure time. The mean overall skin-to-skin time in our series was 92 min. Operating times reported by other authors are higher than in our cohort, ranging from 121, 126 and 135 min respectively [2, 14, 23], to 184 min [10] in comparable case series.

### Software/hardware

All software applications as well as the used printing material must be viewed — according to European Union law — as medical devices. For this reason, we limited ourselves to exclusively using software applications (Materialise Mimics InPrint/ Brainlab iPlan software) and printing material (Formlabs Surgical Guide and Formlabs Dental SG resins) that are already registered as CE-certified class I medical devices in the field of cranio-facial surgery. To use registered medical device software and hardware is more expensive than the free, open-source software but seems to be essential for patient safety and ethical approval of future study concepts.

### Complications

Elective cranioplasty is — in general — associated with higher complication rates than other elective neurosurgical procedures [24]. In a comparable 3D printing-guided cranioplasty case series, Schön et al. also reported a relatively high early reoperation rate in three out of 16 patients (18.75%) because of epidural or subdural postoperative haematomas [23]. As in our series, most of the patients with postoperative haematomas had distinct clinical risk factors for intracranial bleeding after cranioplasty. Ventriculo-peritoneal shunt placement (patient 7) has previously been described as a risk factor [8]. Coagulation disorders like a Factor XIII deficiency (patient 6) or the vital need for early anticoagulation (patient 11) certainly played a crucial role in the postoperative intracranial haematomas that occurred in our series.

### Cost-effectiveness and clinical implementation

The 3D printing and implant modelling workflow we have developed reduce costs of cranioplastic procedures compared to CAD implants and cryoconserved autologous implants. The cost effectiveness of our method is striking, with approximated expenses of around 300 € (= 360 US $) per implant. In contrast, cryoconservation storage of autologous implants can be approximated for our medium size neurosurgical institution, with costs of around 500 € (= 600 US $) per reimplanted bone flap, taking into account several cost drivers (i.e. acquisition costs for freezers, certified packaging material, validation of the freezers at the time of purchase, maintenance and calibration (once a year), and inspection by national health authorities (every 2 years), and costs for microbiological testing) as well as a relatively high rate of autologous bone flaps which must be discarded due to contamination or patient death. CAD implants are available at prices ranging from around 5000 € to 10,000 € (6000–12,000 US $) per implant, signifying a cost reduction with 3D printing-aided cranioplasty of around 95%. This is in line with other studies [14, 23]. The technique could be especially interesting for low-income countries, if CAD implants might not be affordable or available and proper cryoconservation might be difficult.

These economic advantages have triggered the establishment of a 3D printing laboratory at our neurosurgical department. Clinical implementation of the described method has led to a noticeable improvement of implant shaping, operating time and patient satisfaction in our institution. One drawback is that 3D printing is time consuming for the surgeon performing the template design process. However, after a learning phase, we were able to reduce the 3D printing preparation time for a single cranial implant to about 30 min. A further 15 min is needed for postprinting requirements before sterilisation. Printing itself, which takes 3 to 5 h for the template ring and 5 to 8 h for the template mould, can be done any time without the need for constant surveillance.

### Limitations and outlook

The feasibility of the described cranioplasty technique could be demonstrated in a small retrospective cohort. To create more robust data, a prospective, randomised trial with larger cohorts is needed, especially to compare our approach with established implants like CAD implants or autologous bone implants. For now, only straightforward craniectomy defects of the skull have been reconstructed with the “springform” technique. For more complex procedures like skull base reconstructions, the technique does not seem to be suitable for now. Future concepts will aim on 3D printing implants itself without the need for moulding templates.

## Conclusions

A novel approach to design and produce 3D-printed, sterilisable templates to mould custom made PMMA implants for cranioplasty surgery has been developed. The concept of focusing on the outer surface of the implant to achieve the most perfect reconstruction of precraniectomy skull shape has proven to be both simple and cost effective. The clinical implementation of the method shows excellent cosmetic results. The repeated execution of designing and printing processes by operating neurosurgeons may lead to further improvement of the technique in the sense of translational research and might result in further applications of 3D printing in neurosurgery.

## Supplementary Information

Below is the link to the electronic supplementary material.

Supplementary file1 (MOV 335777 KB) Video 1 Preoperative molding of a cranial implant using the “springform”-technique.

## Abbreviations and acronyms

PMMA: Polymethylmethacrylate
CT: Computed tomography
CAD: Computer-aided design
STL: Stereolithography
PreCE: Precraniectomy model
PosCE: Postcraniectomy model
FTP: Fronto-temporo-parietal

## References

[1] Abdel Hay J, Smayra T, Moussa R (2017). Customized polymethylmethacrylate cranioplasty implants using 3-dimensional printed polylactic acid molds: technical note with 2 illustrative cases. World Neurosurgery.

[2] Cheng C-H, Chuang H-Y, Lin H-L, Liu C-L, Yao C-H (2018). Surgical results of cranioplasty using three-dimensional printing technology. Clin Neurol Neurosurg.

[3] da Silva Júnior EB, de Aragão AH, de Paula Loureiro M, Lobo CS, Oliveti AF, de Oliveira RM, Ramina R (2021) Cranioplasty with three-dimensional customised mould for polymethylmethacrylate implant: a series of 16 consecutive patients with cost-effectiveness consideration. 3D Printing in Medicine. 10.1186/s41205-021-00096-7 10.1186/s41205-021-00096-7 PMC7866687 33548008

[4] Feroze AH, Walmsley GG, Choudhri O, Lorenz HP, Grant GA, Edwards MSB (2015). Evolution of cranioplasty techniques in neurosurgery: historical review, pediatric considerations, and current trends. J Neurosurg.

[5] Fiaschi P, Pavanello M, Imperato A, Dallolio V, Accogli A, Capra V, Consales A, Cama A, Piatelli G (2016) Surgical results of cranioplasty with a polymethylmethacrylate customized cranial implant in pediatric patients: a single-center experience. Journal of Neurosurgery: Pediatrics. 10.3171/2015.10.PEDS15489 10.3171/2015.10.PEDS15489 26824593

[6] Fischer CM, Burkhardt J-K, Sarnthein J, Bernays RL, Bozinov O (2012). Aesthetic outcome in patients after polymethyl-methacrylate (PMMA) cranioplasty — a questionnaire-based single-centre study. Neurol Res.

[7] Halani SH, Chu JK, Malcolm JG, Rindler RS, Allen JW, Grossberg JA, Pradilla G, Ahmad FU (2017). Effects of cranioplasty on cerebral blood flow following decompressive craniectomy: a systematic review of the literature. Neurosurgery.

[8] Hirschmann D, Kranawetter B, Kirchschlager C, Tomschik M, Wais J, Winter F, Millesi M, Herta J, Roessler K, Dorfer C (2021). Cranioplasty following ventriculoperitoneal shunting: lessons learned. Acta Neurochir.

[9] Iaccarino C, Kolias AG, Roumy L-G, Fountas K, Adeleye AO (2020). Cranioplasty following decompressive craniectomy. Front Neurol.

[10] Kim B-J, Hong K-S, Park K-J, Park D-H, Chung Y-G, Kang S-H (2012). Customized cranioplasty implants using three-dimensional printers and polymethyl-methacrylate casting. Journal of Korean Neurosurgical Society.

[11] Malcolm JG, Mahmooth Z, Rindler RS, Allen JW, Grossberg JA, Pradilla G, Ahmad FU (2018). Autologous cranioplasty is associated with increased reoperation rate: a systematic review and meta-analysis. World Neurosurgery.

[12] Marbacher S, Andereggen L, Erhardt S, Fathi A-R, Fandino J, Raabe A, Beck J (2012). Intraoperative template-molded bone flap reconstruction for patient-specific cranioplasty. Neurosurg Rev.

[13] Maricevich JBR, Cezar-Junior A, de Oliveira-Junior E, Veras e Silva JM, da Silva JL, Nunes A, Almeida N, Azevedo-Filho HC (2019) Functional and aesthetic evaluation after cranial reconstruction with polymethyl methacrylate prostheses using low-cost 3D printing templates in patients with cranial defects secondary to decompressive craniectomies: a prospective study. Surgical Neurology International. 10.4103/sni.sni_149_18 10.4103/sni.sni_149_18 PMC6357537 30775055

[14] Morales-Gómez JA, Garcia-Estrada E, Leos-Bortoni JE, Delgado-Brito M, Flores-Huerta LE, de La Cruz-Arriaga AA, Torres-Díaz LJ, de León ÁRM-P (2019). Cranioplasty with a low-cost customized polymethylmethacrylate implant using a desktop 3D printer. J Neurosurg.

[15] Mussi E, Mussa F, Santarelli C, Scagnet M, Uccheddu F, Furferi R, Volpe Y, Genitori L (2020). Current practice in preoperative virtual and physical simulation in neurosurgery. Bioengineering.

[16] Oishi M, Fukuda M, Yajima N, Yoshida K, Takahashi M, Hiraishi T, Takao T, Saito A, Fujii Y (2013). Interactive presurgical simulation applying advanced 3D imaging and modeling techniques for skull base and deep tumors. J Neurosurg.

[17] Oliveira AMP, Amorim RLO, Brasil S, Gattás GS, de Andrade AF, Junior FMP, Bor-Seng-Shu E, Iaccarino C, Teixeira MJ, Paiva WS (2021). Improvement in neurological outcome and brain hemodynamics after late cranioplasty. Acta Neurochir.

[18] Panesar SS, Magnetta M, Mukherjee D, Abhinav K, Branstetter BF, Gardner PA, Iv M, Fernandez-Miranda JC (2019). Patient-specific 3-dimensionally printed models for neurosurgical planning and education. Neurosurg Focus.

[19] Piitulainen JM, Kauko T, Aitasalo KMJ, Vuorinen V, Vallittu PK, Posti JP (2015). Outcomes of cranioplasty with synthetic materials and autologous bone grafts. World Neurosurgery.

[20] Pijpker PAJ, Wagemakers M, Kraeima J, Vergeer RA, Kuijlen JMA, Groen RJM (2019). Three-dimensional printed polymethylmethacrylate casting molds for posterior fossa reconstruction in the surgical treatment of Chiari I malformation: technical note and illustrative cases. World Neurosurgery.

[21] Randazzo M, Pisapia J, Singh N, Thawani J (2016). 3D printing in neurosurgery: a systematic review. Surg Neurol Int.

[22] Rosinski CL, Patel S, Geever B (2020). A retrospective comparative analysis of titanium mesh and custom implants for cranioplasty. Neurosurgery.

[23] Schön SN, Skalicky N, Sharma N, Zumofen DW, Thieringer FM (2021). 3D-printer-assisted patient-specific polymethyl methacrylate cranioplasty: a case series of 16 consecutive patients. World Neurosurgery.

[24] Shiban E, Lange N, Hauser A, Jörger A-K, Wagner A, Meyer B, Lehmberg J (2020). Cranioplasty following decompressive craniectomy: minor surgical complexity but still high periprocedural complication rates. Neurosurg Rev.

[25] Tan ETW, Ling JM, Dinesh SK (2016). The feasibility of producing patient-specific acrylic cranioplasty implants with a low-cost 3D printer. J Neurosurg.

[26] Tel A, Tuniz F, Fabbro S, Sembronio S, Costa F, Robiony M (2020). Computer-guided in-house cranioplasty: establishing a novel standard for cranial reconstruction and proposal of an updated protocol. J Oral Maxillofac Surg.

[27] Thiong’o GM, Bernstein M, Drake JM (2021) 3D printing in neurosurgery education: a review. 3D Printing in Medicine. 10.1186/s41205-021-00099-4 10.1186/s41205-021-00099-4 PMC7989093 33759067

[28] Waran V, Narayanan V, Karuppiah R, Owen SLF, Aziz T (2014). Utility of multimaterial 3D printers in creating models with pathological entities to enhance the training experience of neurosurgeons. J Neurosurg.

[29] Winkler PA, Stummer W, Linke R, Krishnan KG, Tatsch K (2000). Influence of cranioplasty on postural blood flow regulation, cerebrovascular reserve capacity, and cerebral glucose metabolism. J Neurosurg.

[30] van de Vijfeijken SECM, Münker TJAG, Spijker R (2018). Autologous bone is inferior to alloplastic cranioplasties: safety of autograft and allograft materials for cranioplasties, a systematic review. World Neurosurgery.

[31] Yeap M-C, Tu P-H, Liu Z-H (2019). Long-term complications of cranioplasty using stored autologous bone graft, three-dimensional polymethyl methacrylate, or titanium mesh after decompressive craniectomy: a single-center experience after 596 procedures. World Neurosurgery.

